# Cranial Bosses of *Choerosaurus dejageri* (Therapsida, Therocephalia): Earliest Evidence of Cranial Display Structures in Eutheriodonts

**DOI:** 10.1371/journal.pone.0161457

**Published:** 2016-08-22

**Authors:** Julien Benoit, Paul R. Manger, Vincent Fernandez, Bruce S. Rubidge

**Affiliations:** 1 Evolutionary Studies Institute (ESI), School of Geosciences, University of the Witwatersrand, Braamfontein, 2050, Johannesburg, South Africa; 2 School of Anatomical Sciences, University of the Witwatersrand, 7 York Road, Parktown, 2193, Johannesburg, South Africa; 3 European Synchrotron Radiation Facility, 71 rue des Martyrs, 38000, Grenoble, France; Royal Belgian Institute of Natural Sciences, BELGIUM

## Abstract

*Choerosaurus dejageri*, a non-mammalian eutheriodont therapsid from the South African late Permian (~259 Ma), has conspicuous hemispheric cranial bosses on the maxilla and the mandible. These bosses, the earliest of this nature in a eutheriodont, potentially make *C*. *dejageri* a key species for understanding the evolutionary origins of sexually selective behaviours (intraspecific competition, ritualized sexual and intimidation displays) associated with cranial outgrowths at the root of the clade that eventually led to extant mammals. Comparison with the tapinocephalid dinocephalian *Moschops capensis*, a therapsid in which head butting is strongly supported, shows that the delicate structure of the cranial bosses and the gracile structure of the skull of *Choerosaurus* would be more suitable for display and low energy combat than vigorous head butting. Thus, despite the fact that *Choerosaurus* is represented by only one skull (which makes it impossible to address the question of sexual dimorphism), its cranial bosses are better interpreted as structures involved in intraspecific selection, i.e. low-energy fighting or display. Display structures, such as enlarged canines and cranial bosses, are widespread among basal therapsid clades and are also present in the putative basal therapsid *Tetraceratops insignis*. This suggests that sexual selection may have played a more important role in the distant origin and evolution of mammals earlier than previously thought. Sexual selection may explain the subsequent independent evolution of cranial outgrowths and pachyostosis in different therapsid lineages (Biarmosuchia, Dinocephalia, Gorgonopsia and Dicynodontia).

## Introduction

Permo-Triassic Therapsida, particularly well known from the Karoo rocks of South Africa, form the stem group of Mammaliaformes that eventually gave rise to mammals [1–3]. Extant mammals are characterised by a relatively large brain, compared to non-mammalian and non-avian vertebrates, that enables a broad range of behaviours (e.g. ritualized displays, behavioural adaptations to environmental changes, and sociality, amongst others) [2, 4–6]. Thus an understanding of the potential behavioural repertoire of early therapsids may elucidate deep evolutionary roots of mammalian behaviour and the neuroanatomy that enabled these behaviours [2, 6–9].

Analyses of anatomical injuries, fossilised tracks and nests, and uniquely preserved fossil evidence of behaviour “caught in action”, allow us to infer the behaviour of extinct species based on what is known from extant forms [10–14]. In addition, speculation regarding the behaviour of fossil species has been inferred from conspicuous osseous structures that are directly related or dedicated to a certain type of behaviour in extant animals, such as cranial bosses or hornlike structures for intraspecific agonistic behaviour [10, 15–18].

Cranial ornamentation is not rare in the therapsid fossil record. By the early Permian, cranial bosses were already present in the basal therapsid *Tetraceratops* [19], and have been reported in many dicynodonts [20, 21, 22], dinocephalians [15, 23–27], Gorgonopsians [28, 29], and biarmosuchians [1, 30–32]. Their function is rarely discussed, but they are sometimes interpreted as sexually dimorphic traits [33–37] or as weapons for head to head intraspecific combat [15, 22, 34]. Head-butting behaviour in Dinocephalia was first hypothesized by Brink [23], later demonstrated by Barghusen [15], and is now generally accepted (e.g. [1, 3]). Geist [38] hypothesized much the same behaviour for some dicynodonts. Though less widely acknowledged, this hypothesis has been proposed for some sexually dimorphic dicynodonts, such as *Diictodon*, *Pelanomodon* or *Placerias* [22, 34, 39]. Also, the enlarged canines of the basal anomodont *Tiarajudens eccentricus* have been interpreted as a possibly sexually dimorphic structure for intraspecific combat or intimidation [9].

**Fig 1:**
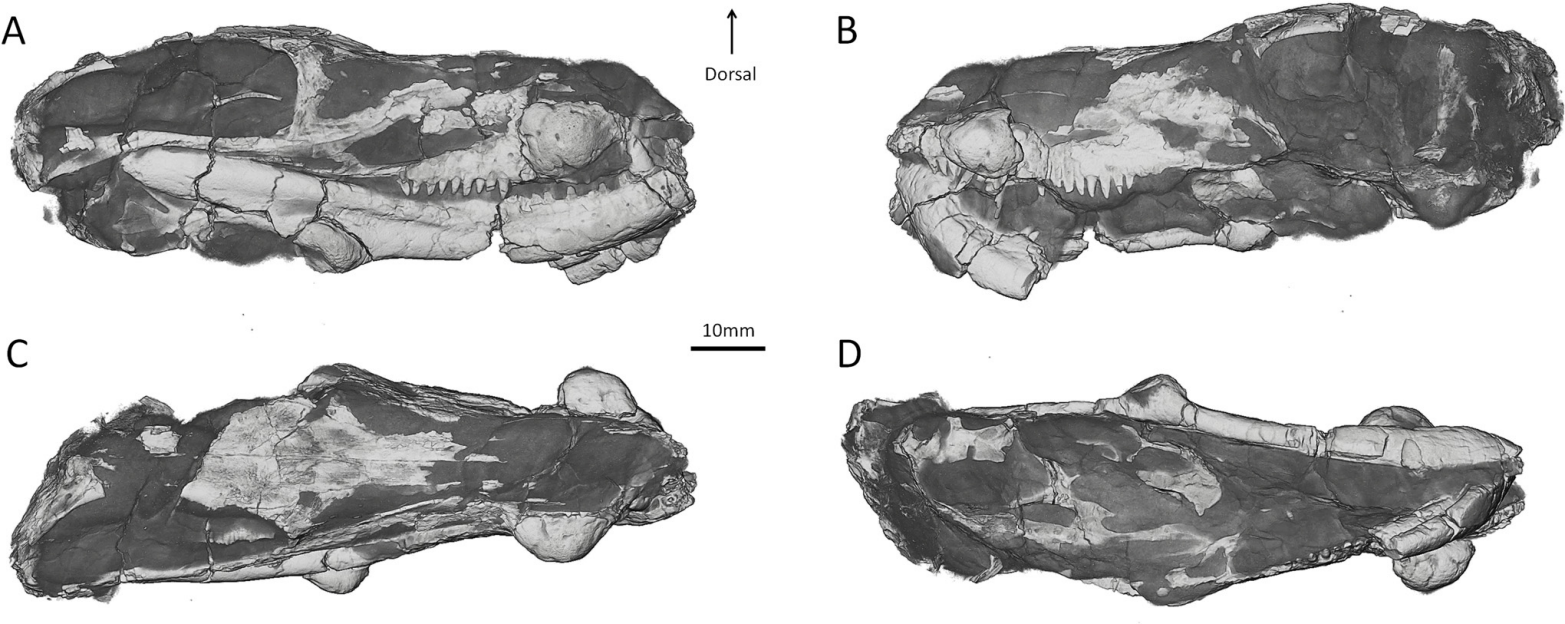
Digital 3D rendering of the skull of *Choerosaurus dejageri*. A, lateral right view; B, lateral left view; C, dorsal view; D, ventral view. The matrix appears in shades of dark grey and the preserved bone is in light grey.

Cranial ornamentation in non-mammaliaform therapsids thus presents an excellent opportunity to assess behaviour at the root of the mammalian evolutionary tree. Among Permo-Triassic Eutheriodontia (Therocephalia and Cynodontia, the closest relatives of mammaliaforms amongst non-mammaliaform therapsids [1, 3]) the enigmatic therocephalian *Choerosaurus dejageri* is the only theriodont species to have conspicuous cranial outgrowths. *Choerosaurus dejageri* is known from only the holotype (SAM-PK-K 8797) from Kuils Poort Farm (Beaufort West, South Africa) and is housed at Iziko Natural History Museum (Cape Town, South Africa) [40]. The skull has two paired symmetrical bosses on the maxilla and mandible (Fig 1), but apart from the original description by Haughton [40] little research has been undertaken on the specimen. In his work, Haughton described the specimen as one of the Scaloposauridae. More recent authors (e.g. [41–43]) reassigned it to the Lycideopidae, a more derived family of baurioid therocephalians, based on the gracile aspect of the skull and its advanced dentition. The conspicuous maxillary and mandibular outgrowths are unique to *Choerosaurus* among eutheriodonts and have not yet been described in detail or assessed functionally.

Given the importance of cranial bosses for observable sexual dimorphism, intraspecific competition, sexual selection, species recognition and social interaction (male-male combat, display and ritualized courtship) leading to access to mates or to secure territory [44, 45], a thorough investigation of these structures in *Choerosaurus* provides a unique opportunity to shed new light on the evolution of eutheriodont behaviour and the evolutionary roots of mammalian sociality.

Due to the delicate and unique nature of specimen SAM-PK-K 8797, computerized X-ray microtomography (microCT scan) was used to reveal the internal cranial morphology of *Choerosaurus*. Cranial bosses are usually associated with agonistic behaviour (head and flank butting), and this affects the internal structure of the skull [15, 17, 18, 46]. Hornlike structures are also involved in a variety of intimidation behaviours, and can be used as adornments for mate attraction, or as resonating chambers to produce sounds (e.g. the hollow horns of *Arsinoitherium* and lambeosaurin dinosaurs), resulting in them having a specific internal osseous structure [44, 45, 47, 48]. Thus, the internal structure of the cranial outgrowths, which can be reconstructed using CT scans, can provide information on the potential function of these outgrowths. Based on CT data the internal osseous structure of the maxillary and mandibular bosses of *Choerosaurus dejageri* was compared with that of the cranial roof of the dinocephalian *Moschops capensis*, a Permian therapsid in which head butting is strongly supported [15]. Our aim was to assess whether the cranial outgrowths of *Choerosaurus* were used as a weapon for intraspecific combat or whether they were ornamental.

## Material and Methods

Specimen SAM-PK-K 8797 is a complete skull of the lycideopid therocephalian *Choerosaurus dejageri* (*Tropidostoma* Assemblage Zone of the Beaufort Group, South Africa, ~259 Ma) housed in the Iziko Museum of Natural History (Cape Town, South Africa). It was scanned at the CT scanner Unit of the Central Analytical Facility at the University of Stellenbosch (South Africa) using a General Electric Phoenix VTomeX L240 micro-CT scanner with a isotropic voxel size of 50 microns.

Specimen AM6556 is the complete skull of a juvenile tapinocephalid dinocephalian *Moschops capensis* (*Tapinocephalus* Assemblage Zone of the Beaufort Group, South Africa, ~265 Ma) housed in the Albany Museum (Grahamstown, South Africa). The brain case of this skull was scanned at the ID17 beamline of the European Synchrotron Radiation Facility (ESRF, Grenoble, France; proposal ES339). The setup consisted of a FReLoN-2eV camera, a 0.5x magnification set of lenses, a 2 mm LuAG scintillator, a monochromatic X-ray beam of 150 keV (bent double-Laue crystals) and a sample-detector distance of 10.9 m to perform Propagation Phase Contrast Synchrotron micro Computed Tomography (PPC-SRμCT). The tomography was computed based on 2510 projections (58 x 1024 pixels, binning factor of 2) of 0.5 s each over 360 degrees resulting in data with a 117.23 μm isotropic voxel size. An attenuation protocol [49] allowed an increase in the exposure time, to compensate for X-ray attenuation by the sample, without saturating the detector. Additionally, the center of rotation was shifted by ~35 mm to increase the horizontal field of view in the reconstructed data (i.e., half acquisition protocol). Given the limited vertical field of view, 50 scans were necessary (30% of vertical overlap between two consecutive scans) to cover the full height of the sample. The tomographic reconstruction was performed using the single distance phase retrieval approach of the software PyHST2 [50, 51]. The resulting 32 bits data were converted to a stack of 16 bits tiff using the min and max crop values from the 3D histogram generated by PyHST2.

Three-dimensional renderings of the internal structure of the maxillary canal of *Choerosaurus* and the braincase of *Moschops* were obtained using manual segmentation under Avizo 8 (FEI VSG, Hillsboro OR, USA). Unlike [52] where only the parts of the canal that directly communicate with the external surface were segmented, all parts of the maxillary canals were segmented. All measurements, CT images and 3D rendering were obtained using Avizo 8 (VSG).

## Description

### Maxillary and mandibular bosses of *Choerosaurus dejageri*

**Fig 2:**
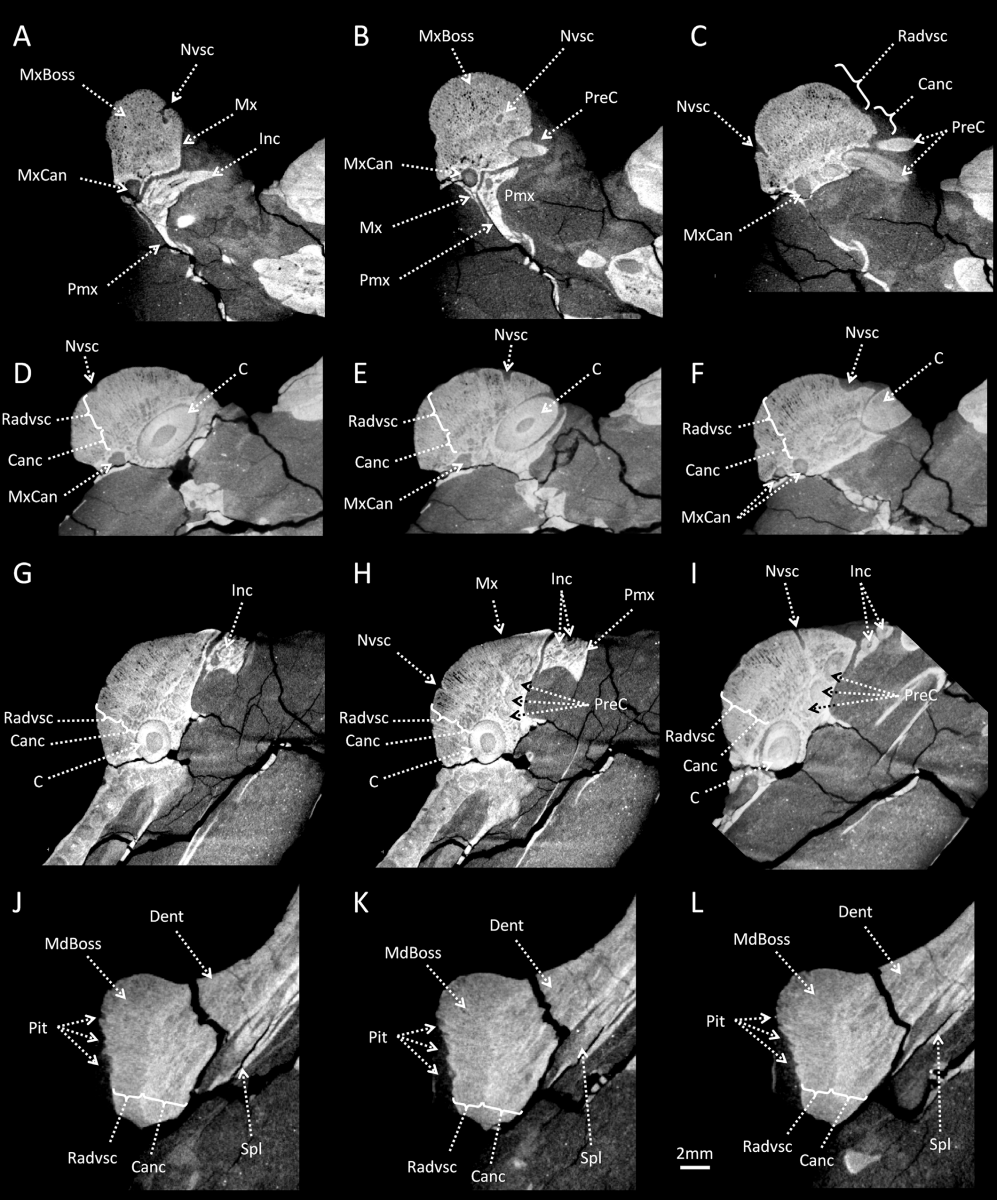
CT images of the maxillary and mandibular bosses of *Choerosaurus dejageri*. A-F, the maxillary boss in transverse sections. G-I, the maxillary boss in longitudinal sections. J-L, the mandibular boss in longitudinal sections. Abbreviations: C, Canine; Canc, Cancellous bone; Dent, Dentary; Inc, Incisor; MdBoss, Mandibular boss; Mx, Maxilla; MxBoss, Maxillary boss; MxCan, Maxillary canal; Nvsc, Neurovascular canal; Pit, Pitted surface of the mandibular boss; Pmx, Premaxilla; PreC, Precanine tooth on the maxilla; Radvsc, Radial vasculature; Spl, Splenial. Scale bar: 2 mm.

**Fig 3:**
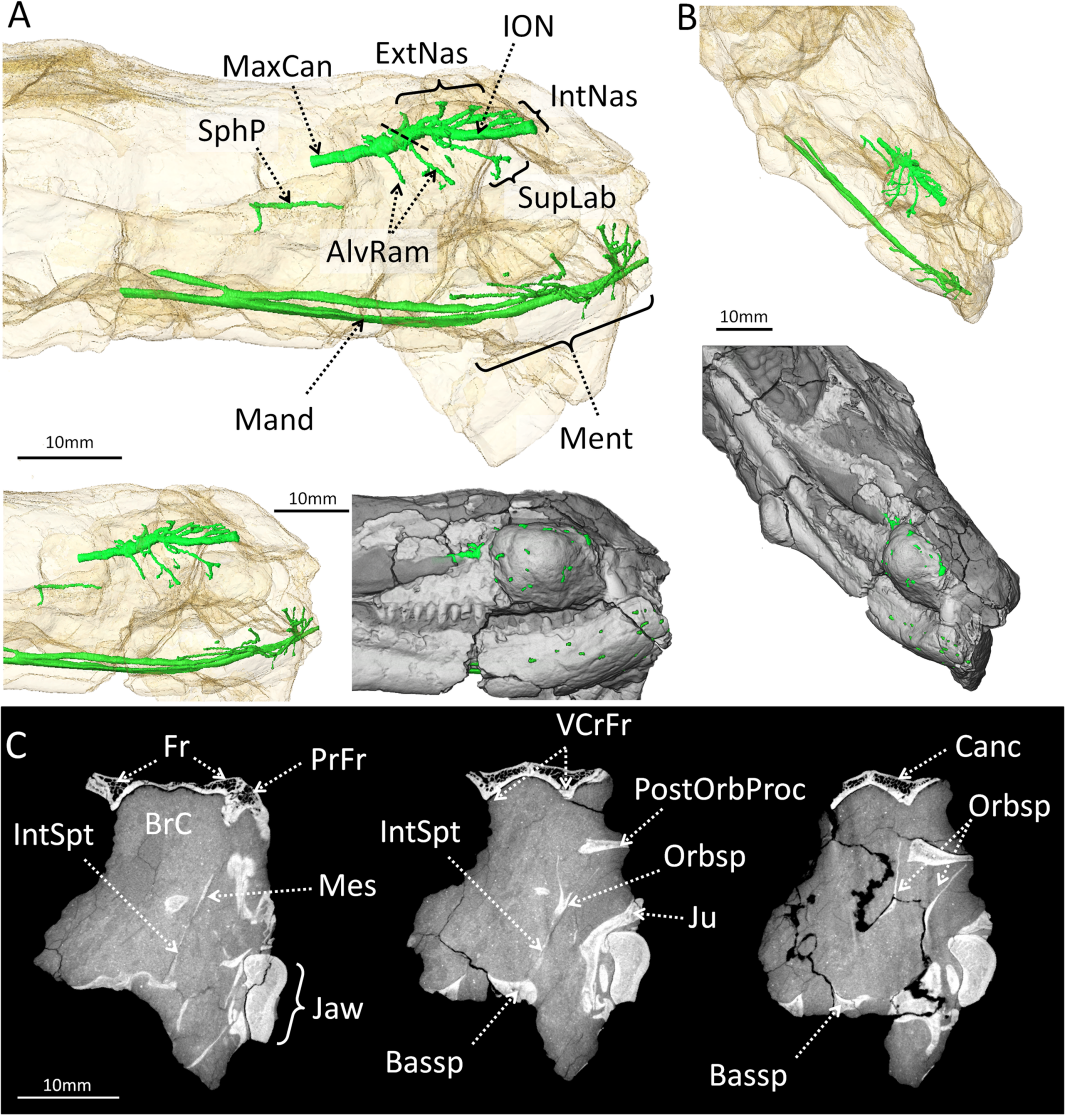
Three-dimensional reconstruction of the maxillary canal, mandibular canal, sphenopalatine canal and brain-case of *Choerosaurus dejageri*. A, Lateral view with the skull transparent (top) and non-transparent (below). B, oblique view with the skull transparent (top) and non-transparent (below). The trigeminal nerve occupies the majority of these canals [52, 53], and the identification of trigeminal branches is based on the name of the corresponding nerve in mammals. C, CT images of the transverse section throught the braincase of *Choerosaurus dejageri* (from left to right: rostral to caudal sections). Abbreviations: AlvRam, alveolar rami; Bassp, basiosphenoid; BrC, braincase; Canc, cancellous bone; ExtNas, external nasal rami of the infraorbital nerve; Fr, frontal; IntNas, internal nasal rami of the infraorbital nerve; IntSpt: interorbital septum; ION, infraorbital nerve; Jaw, lower jaw bones; Ju, jugal; Mand, mandibular rami; Mes, mesethmoid; Ment, mental foramina; MxCan, maxillary canal; Orbsp, loose orbitosphenoid; PrFr, prefrontal; PostOrbProc, loose postorbital process; SphP, sphenopalatine rami; SupLab, supralabial ramus of the infraorbital nerve; VCrFr, ventral crests of the frontal. Scale bar: 10 mm.

A hemispheric maxillary boss is present on either side of the maxilla. The right maxillary boss is 14 mm long and 11 mm high, while the left is 12 mm long and 9 mm high. The left maxillary boss is broken and has been glued to the maxilla, whereas the right boss is intact. As such, our description is based mainly on the right side of the specimen, which shows the best preservation (Fig 1). Although positioned rostrally, just posterior to a distinct suture separating the maxilla and premaxilla (Fig 2A–2C), the maxillary boss is formed entirely by the maxilla. The premaxilla bears seven incisors on the left side but only two are preserved on the right (see S1 Video and S2 Video), and the maxilla has three precanine teeth (Fig 2G–2I). The maxillary boss is located above the last incisor, the precanines, the canine and the first postcanine tooth (Figs 1 and 2A–2I). The boss is not hollow and is filled with two types of spongious bone tissue that are distinct from the compact bone forming the maxilla (Fig 2A–2I). Proximally, the core of the boss comprises one to two millimeters of cancellous bone with large hollow spaces (Fig 2D–2I). The external body of the boss comprises denser bone that contains an abundance of radially oriented, presumably vascular, canals that result in the surface of the maxillary boss having a rough texture (Fig 2D–2I). In addition a dozen larger neuro-vascular foramina also perforate the maxillary boss (Fig 2A–2C, 2E, 2I and Fig 3A and 3B). These foramina lead to canals that penetrate the maxillary boss to join the maxillary canal.

In therapsids, the maxillary canal houses the maxillary nerve and vessels as well as a branch of the facial nerve [52]. In *Choerosaurus*, the maxillary canal has a special relationship with the maxillary boss since most of its ramifications open distally on the surface of the boss (Fig 3A and 3B). The main branch of maxillary nerve extends inside the maxilla along the ventral half of the maxillary boss (Figs 2A–2F and 3) and manifests all the usual branches of this canal [52]. Caudally, the maxillary canal ramifies into two branches for the alveolar rami (Fig 3A and 3B). The infraorbital ramus begins anterior to the bifurcation of the alveolar rami. Rostral to this point, three canals for the external nasal rami bifurcate dorsally (Fig 3A and 3B). Ventrally, two other canals for the superior labial rami bifurcate at approximatelly the same level (Fig 3A and 3B). The maxillary canal terminates anteriorly as three inferior nasal rami (Fig 3A and 3B). Except for the main trunk which ends rostrally to the maxillary boss, all the branches open externally in foramina located on the surface of the maxillary boss (Fig 3A and 3B).

The mandibular boss is located on the postero-ventral angle of the dentary (Figs 1A and 2J–2L). There is only one mandibular boss, preserved on the right side (9 mm long and 7 mm high), with the presumed contralateral boss having not been preserved in this specimen. It is not fully hemispheric, like the maxillary bosses, as it is flattened antero-dorsally (Fig 1A). The internal structure of the mandibular boss is denser than that of the maxillary bosses. The radial pattern of the external body of the mandibular boss is less pronounced than that of the maxillary bosses, perhaps because of the smaller size of the vascular canals (Fig 2J–2L). The presence of these canals is evidenced by pits that create irregularities on the surface of the boss (Fig 2J–2L), but there is no evidence for large neuro-vascular canals connected to the mandibular canal for the mandibular branch of the trigeminal nerve. Instead, the mandibular canal runs inside the dentary and ramifies only at the extremity of the mandible to supply the mental foramina (Fig 3A and 3B). The core of the boss is cancelleous like that of the maxillary boss (Fig 2J–2L).

### Structure of the braincase of *Choerosaurus dejageri*

Few remnants of the braincase of *Cheorosaurus* are preserved, primarily because the braincase is generally not well ossified in non-mammalian eutheriodonts. In these taxa, ossification of the braincase is usually limited to its dorsal aspect, the ventral side being ossified only posterior to the pituitary fossa by the basicranium [54, 55]. In some cases, the region of the forebrain can be partially ossified by a short and often loose gutter formed by the sphenethmoid complex, but it still leaves the region of the olfactory bulbs and a wide gap between the sphenethmoid and basicranium unossified [54, 55]. Additionally, the braincase walls are not well preserved in SAM-PK-K 8797. For instance, the parietal bones are missing and the basicranium is crushed (Fig 1). Nevertheless, it can be ascertained, based on what is preserved, that in contrast to the condition in *Moschops* (see below), the walls of the braincase are not thickened in *Choerosaurus* (Fig 3C). The braincase is delicately built, limited dorsally by the thin and cancellous frontal bone (Fig 3C). The presence of a very thin sphenethmoid complex, found loose in the braincase of specimen SAM-PK-K 8797 (Fig 3C), indicates that a segment of the rostral part of the braincase was ventrally ossified in *Choerosaurus*. The sphenethmoid complex is formed by the fusion of the mesethmoid (rostrally), the orbitosphenoid (caudally), and the interorbital septum (ventrally) (Fig 3C). Sutures are indistinct on the CT scan. Rostrally, the orbitosphenoid appears Y-shaped in cross section and then the two ascending laminae diverge posteriorly into two distinct, symmetrical bony walls (Fig 3C). The ascending laminae of the orbitosphenoid may have articulated dorsally with the corresponding ventral crests on the frontal bones to enclose the braincase in a gutter-like structure (Fig 3C). Caudally, the basicranium is too deformed and disarticulated to address the morphology of the hindbrain. Despite the fact that Haughton [40] described a parietal foramen in *Choerosaurus*, this region of the skull is not preserved and thus, there is no remnant of the parietal foramen (Fig 1, S1 Video).

### Structure of the fronto-parietal shield and braincase in *Moschops capensis*

**Fig 4:**
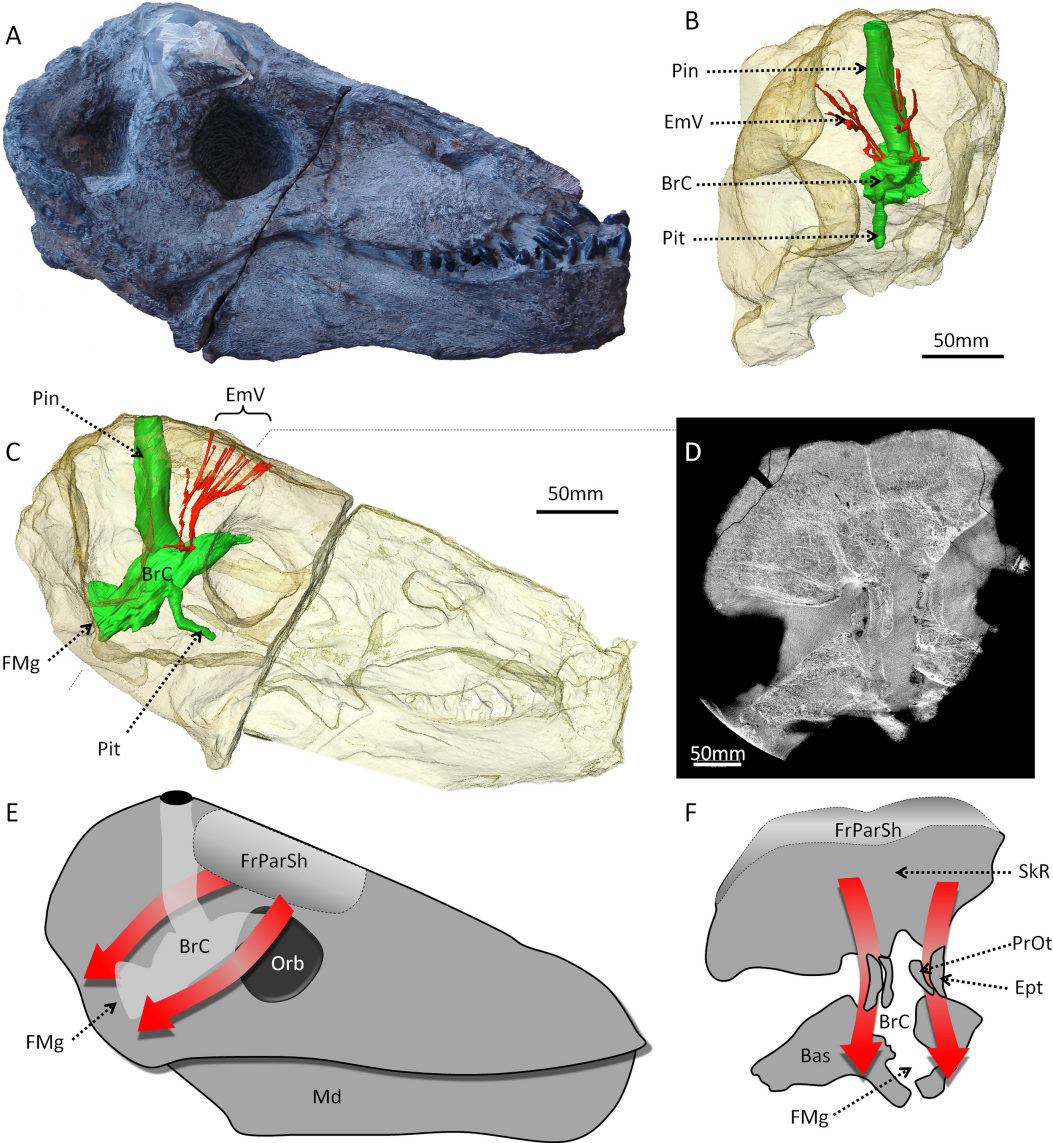
The skull and braincase of *Moschops capensis*. A, the skull of *Moschops* in lateral view. B-C, 3D rendering from CT data, B, the endocranial cast (green) and emissary vein (red) of *Moschops* in frontal view. C, the endocranial cast (green) and emissary vein (red) of *Moschops* in lateral view. D, virtual transverse section through the braincase of *Moschops*. E-F, the pathway of the shockwave (red arrows) thourgh the braincase of *Moschops* illustrated on the lateral view (E) and the transverse section (F). Abbreviations: Bas, basicranium; BrC, Braincase; EmV, emissary veins; Ept, Epipterygoid; Fmg, foramen magnum; FrParSh, Fronto-parietal shield; Md, mandible; Orb, orbit; Pin, pineal tube; Pit, pituitary fossa; PrOt, prootic; SkR, skull roof. Scale bar: 50mm.

**Fig 5:**
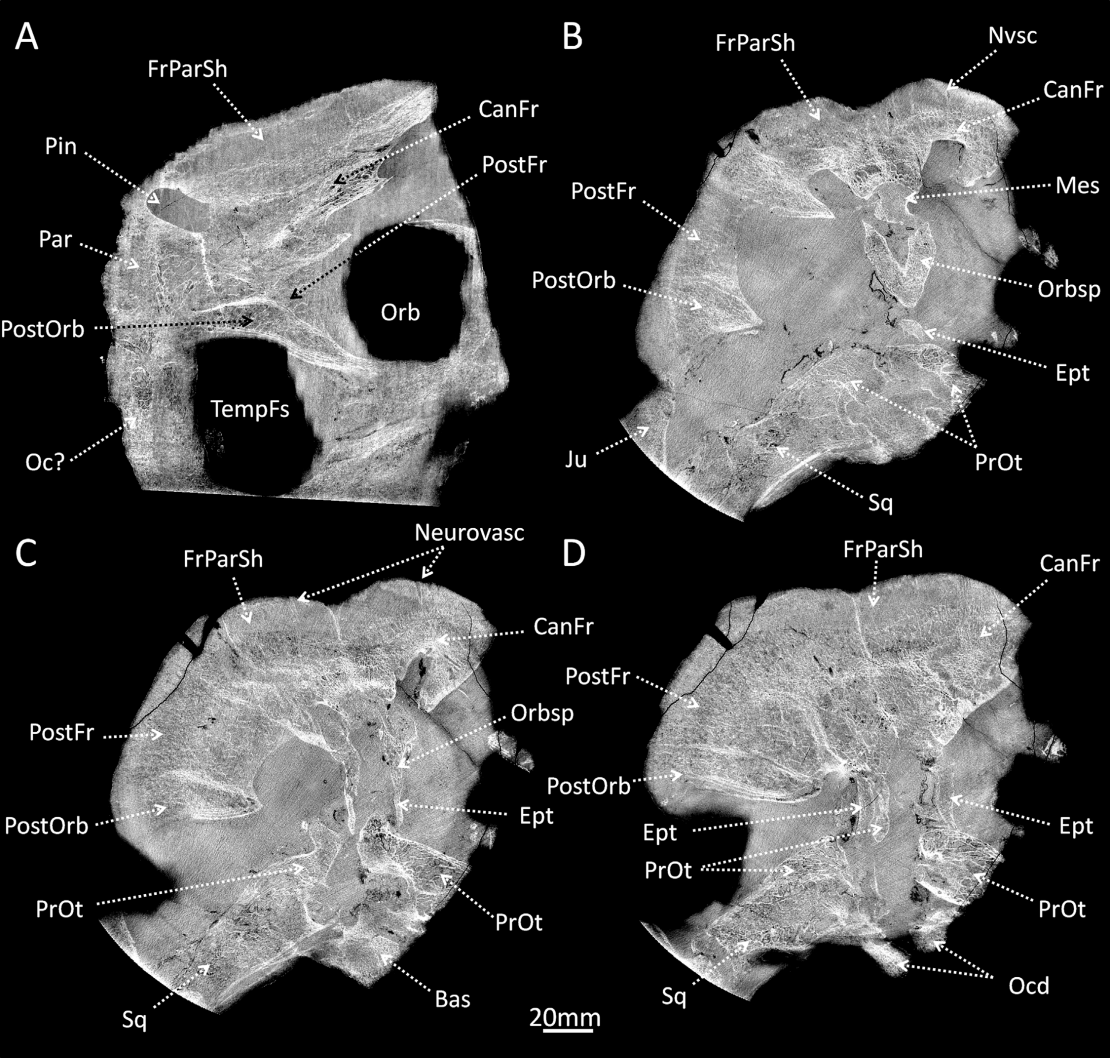
CT images of the braincase of *Moschops capensis* AM6556. A, longitudinal section. B-D, transverse sections. Abbreviations: Ept, epipterygoid; CanFr, cancellous frontal bone; FrParSh, frontoparietal shield; Ju, jugal; Mes, mesethmoid; Nvsc, neurovascular canal; Oc?, unidentified occipital bone; Ocd, occipital condyle; Orb, orbite; Orbsp, orbitosphenoid; Par, Parietal; Pin, pineal tube; PostFr, postfrontal; PostOrb, postorbital; PrOt, prootic; Sq, squamosal; TempFs, temporal fenestra. Scale bar: 20mm.

The skull of tapinocephalid dinocephalians is robustly built, seemingly to accommodate direct impacts on the cranial vault as a result of head butting [15]. Thus, the bones forming the post-orbital bar, the temporal arch, and the skull roof (dermatocranium) are thickened to absorb and ameliorate the effect of blows during combat, and the braincase is rotated backwards so that the foramen magnum is aligned ventrally to the fronto-parietal shield (Fig 4). This allows for the energy of the potential blows to be directly transmitted to the vertebral column [15, 23] (Fig 4E and 4F). As a result of the development of the fronto-parietal shield, and the re-orientation of the braincase, the pineal foramen is also caudally deflected (Fig 4C and 4E). The fighting shield comprises mainly the frontal bones, and all bones involved in the cranial vault and the braincase are pachyostotically thickened (Fig 5). At the level of the braincase, the thickness of the cranial vault varies from 50 to 60 mm in cross section (Fig 5B–5D). The orbitosphenoid, epipterygoid and prootic are also pachyostotic, with a thickness of approximately 5 mm as determined from CT slices, which result in the almost complete ossification of the braincase (Fig 5B–5D). Amongst synapsids, complete ossification of the braincase is documented only in dinocephalians and mammaliaforms [2, 24, 54].

In addition to their thickness, the surfaces of the parietal and frontal bones comprise 15–20 mm of dense osteosclerotic bone forming the fronto-parietal shield (Fig 4D and 4F and Fig 5). Our scan resolution is too low to determine whether this layer of dense bone contains radially oriented vascular canals as in the bosses of *Choerosaurus* or the cranial dome of pachycephalosaurids [17, 18]; however, some neuro-vascular canals penetrate deep inside the frontal and parietal bones to end inside the cancellous tissue (Fig 5B and 5C) and may have supplied a cornified plate that covered the fronto-parietal shield [15]. Some of the larger canals join the braincase and thus may represent emissary veins (Fig 4B and 4C). The fronto-parietal shield is histologically divided into a central core of cancellous bone and an outer shell of dense bone (Figs 4F and 5). This division possibly enabled greater robustness of the shield during blows while also minimizing the mass of the skull. Also, the cancellous tissue could have acted like the frontal sinus in bovids in absorbing and dissipating loads applied to the fronto-parietal shield [44, 46]. As specimen AM6556 is a young individual, there could be another, ontogenetic explanation. In juvenile pachycephalosaurid dinosaurs a zone of cancellous bone underneath the cranial dome is resorbed during growth [17] and a similar phenomenon could have applied to dinocephalians; however, in pachycephalosaurids this internal zone corresponds to a rapidly growing region of the skull and the vascular tissue in this zone is arranged radially [17]. This is not evidenced in our specimen of *Moschops* (Fig 5) which would give support to the functional interpretation of the presence of these cancellous bones (but note that data are missing for the palaeohistology of cranial bones in Dinocephalia). Finally, although the *Moschops* cranial vault and braincase are pachyostotic, the bones not directly involved in the fronto-parietal shield (such as the base of the frontal, post-frontal, post-orbital and prootic) are particularly rich in large pneumatic spaces (Fig 5).

## Discussion

The function of the maxillary and mandibular bosses in *Choerosaurus dejageri* has not been discussed previously, and as this species is among the only Permo-Triassic eutheriodont to have prominent bony outgrowths an assessment of their bearing on the evolution of eutheriodont behaviour is of interest. The fact that both maxillary bosses are symmetrically present on either side of the skull indicates that they are not pathological. The mandibular boss is preserved on one side only because the left ramus of the mandible is not present, but it is likely that this structure was symmetrical given i) that the maxillary bosses are present on both sides and ii) since a similar, yet smaller, boss is symmetrically present on the angle of the dentary in the closely related karenitid therocephalians [42]. The radial vasculature pattern in the *Choerosaurus* bosses suggests that they were fast growing structures that would have required a considerable investment of energy [17, 18, 56–58]. In order to be maintained by natural selection, such an allocation of energy must bring a substantial benefit to general fitness. As such, to be retained by natural selection their presence must have been beneficial for either survival and/or reproduction. It is thus probable that these cranial bosses were involved in an important function. Here we first discuss the adaptations of the skull roof and braincase of *Moschops capensis* to head butting in order to address if similar adaptations are present in *Choerosaurus dejageri* which would support fighting behaviour. The implications of these comparisons on the evolution of sexually selected traits are then discussed.

### Adaptation to head butting in *Moschops capensis*

Head to head confrontation in *Moschops* is accompanied by a suite of derived traits to reinforce the skull and to protect the central nervous system (e.g. thickened post-orbital bar, posteriorly inclined occipital surface and presence of a protruding and roughened surface on the fronto-parietal shield that may have supported a cornified plate, see [15]). In addition to the features previously recognised by [15], *Moschops* has a dramatically thickened cranial vault (Fig 5), a fronto-parietal shield comprising dense bone tissue (Figs 4F and 5), and a completely ossified braincase that includes columnar and pachyostotic orbitosphenoid and epipterygoid complexes to protect the brain and transfer shock waves to the base of the skull (Fig 4E and 4F and Fig 5). The presence of cranial outgrowths and pachyostosis in adults is a unifying character of dinocephalians [26, 28, 59]. To date an ossified sphenethmoidal complex completely surrounding the braincase in non-mammaliaform therapsids has been documented only in anteosaurids, titanosuchids and tapinocephalids [24], but because these internal structures are masked by the skull roof most dinocephalian descriptions lack data on the degree of ossification of the braincase (e.g. [25, 26, 60–62]). The reported absence of the orbitosphenoid and other sphenoidal elements in the basal anteosaurid *Sinophoneus* [56], suggests that braincase ossification occurred only among more derived dinocephalians.

In *Moschops*, the entire braincase is rotated posteriorly so that its rostral end points dorsally when the skull is orientated horizontally (Fig 4C) [23, 24, 28]. This backward rotation results in a posteriorly shifted parietal foramen. As a zone of weakness in the architecture of the braincase, the parietal foramen is thus re-located on the caudal margin of the cranial roof, away from the fronto-parietal shield so that the skull roof is not weakened (Fig 4E). Among the dinocephalians, the basal genera *Sinophoneus*, *Estemmenosuchus*, *Syodon*, *Notosyodon* and *Australosyodon* display a more anteriorly located parietal foramen [25, 26, 60–62]. In these five genera the anterior wall of the pineal boss is formed by both the frontal and parietal bones whereas it is formed by only the parietal in *Doliosauriscus*, *Titanophoneus* and other more derived dinocephalians [25, 26, 60–62]. This could indicate less extreme head butting in the basal genera which have less cranial outgrowths and pachyostosis [26, 27].

### Did the *Choerosaurus* bosses function as a weapon?

**Fig 6:**
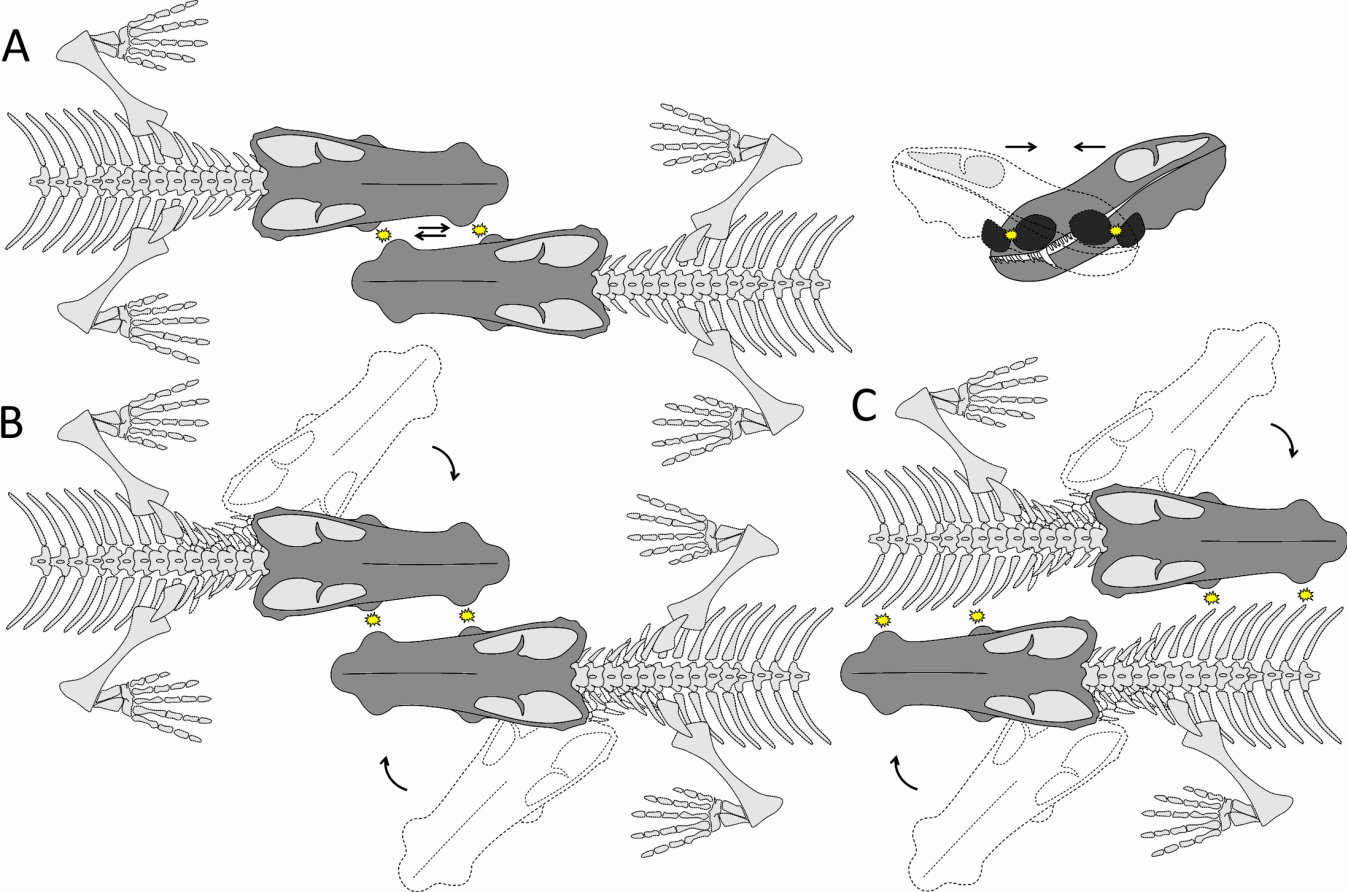
Different types of fighting hypothesized in *Choerosaurus dejageri*. A, lateral head pushing; B, lateral head butting; C, lateral flank butting. The arrows represent head movements. The stars represent the points of impact.

Some of the pachyostotic characteristics of dinocephalians are also present in burnetiamorph biarmosuchians which have a variety of prominent ‘horns’ on their skulls, particularly above and between their orbits, and in the squamosal region [1, 30–32, 63–67]. This strongly suggests that burnetiamorph biarmosuchians and dinocephalians convergently evolved their heads as a weapon. In particular the highly pachyostosed burnetiamorph *Pachydectes* has a pair of maxillary bosses similar to those of *Choerosaurus* [31]. The surface of the maxillary boss in *Pachydectes* has a pitted texture reminiscent of that of *Choerosaurus* and suggests a similar radial pattern organisation of the maxillary boss vasculature. These similarities suggest that *Choerosaurus* used its facial outgrowths as weapons against predators and/or during intraspecific fights either in direct head to head or head to flank combats (Fig 6).

This hypothesis is supported by the fact that the maxillary boss of *Choerosaurus* is well innervated and vascularized (Figs 2A–2I and 3) and its roughened external surface, with numerous neuro-vascular foramina (Fig 3A and 3B), is similar to that of a giraffe ossicone [68] and may have been covered by skin or a cornified sheath [69]. It is now well established that male giraffes use their ossicones to club each other during rutting, flank-butting combat [70].

However, there are some caveats to this hypothesis. First, the gracile and lightly built cranial structure of *Choerosaurus*, with its extensive interpterygoid vacuities and its thin, possibly incompletely ossified post-orbital bar [40, 41], make the use of the head in direct combat unlikely. In *Moschops*, where cranial pachyostosis is an important element for head butting as it keeps the brain safe from injuries, the skull roof comprises 50 to 60 mm of thickened bone, of which about 5 mm is osteosclerotic at the level of the fronto-parietal shield. No such thickening or reinforcement is present in the skull of *Choerosaurus* which comprises mostly thin cancellous bone, even in the portion of the maxilla and mandible located close to the bosses (Fig 2A–2C and 2J–2L). Also, the nervous system of *Choerosaurus* is not protected against shock. In *Moschops*, the orbitosphenoid and epipterygoid complexes form columnar bones (with a thickness of about 5 mm) that probably aided in transmiting shock waves through the braincase to the vertebral column, thus protecting the central nervous system (Fig 4D–4F). In contrast the cranial vault and orbitosphenoid of *Choerosaurus* are very thin (Fig 3C) and apart from the presence of the bosses, the skull of *Choerosaurus* is as gracile as that of its non-specialized close therocephalian relative *Tetracynodon* [41]. It is important to note that not all extant head-butting species have a skull as well adapted as that of *Moschops* for high energy frontal ramming and/or head butting [15]. Conversely, differing degrees of violence are employed amongst extant mammals, ranging from low energy wrestling, flank butting or lateral thrusts, to high energy frontal pushing and ramming [44, 45]. *Choerosaurus* displays no obvious cranial pachyostosis or protection of the central nervous system which excludes high energy combat, but this does not exclude the use of the head in some forms of low energy combat since some bovids, such as the mountain goat *Oreamnos*, use their delicate and fragile head for flank fighting [44]. Therefore, *Choerosaurus* may have been able to engage in low energy combats, like flank butting (Fig 6C).

As indicated earlier, because the maxillary canal opens directly onto the surface of the maxillary boss, the somatosensory fibres of the trigeminal nerve of *Choerosaurus* would not have been protected (Fig 3A and 3B), and would have been directly exposed to the impacts. If the maxillary boss was used for fighting, this would have resulted in neural injury. It would be expected that a structure directly involved in combat would not be densely innervated and less vascularized to prevent excessive bleeding and pain, as exemplified by the fronto-parietal shield of *Moschops* (Fig 4B and 4C). In addition, the ramifications of the maxillary canal running through the maxillary boss of *Choerosaurus* weaken its internal structure (Figs 2A–2I and 3) making its use as a weapon even more unlikely. In contrast, the mandibular boss of *Choerosaurus* appears as a better candidate to serve as a structure used in combat because it is denser and vascularized by smaller and more tightly arranged canals, without any large neuro-vascular canal to weaken its structure (Fig 2J–2L). In this respect, it is noteworthy that the angular bone on the jaw of anteosaurid dinocephalians bear flattened and dense bosses that are reminiscent of the dentary bosses of *Choerosaurus* [27].

Histologically, the maxillary and mandibular bosses of *Choerosaurus* are similar as they display the same radial pattern of vascular canals. This radial arrangement of the vascular canals in the bosses could be interpreted as an adaptation for agonistic combat (e.g. [71]) as a similar radial pattern of fibrolamellar bone is also present in the frill of ceratopsid [56] and in the dome of pachycephalosaurid dinosaurs [17, 18]. A similar pattern is possibly present in the dinocephalian *Moschops* and in burnetiamorph biarmosuchians (see above). However, this kind of radial arrangement of the vasculature results from the rapid deposition of osseous tissue during development and is therefore not necessarily direct evidence for head butting [17, 18, 56–58].

**Fig 7:**
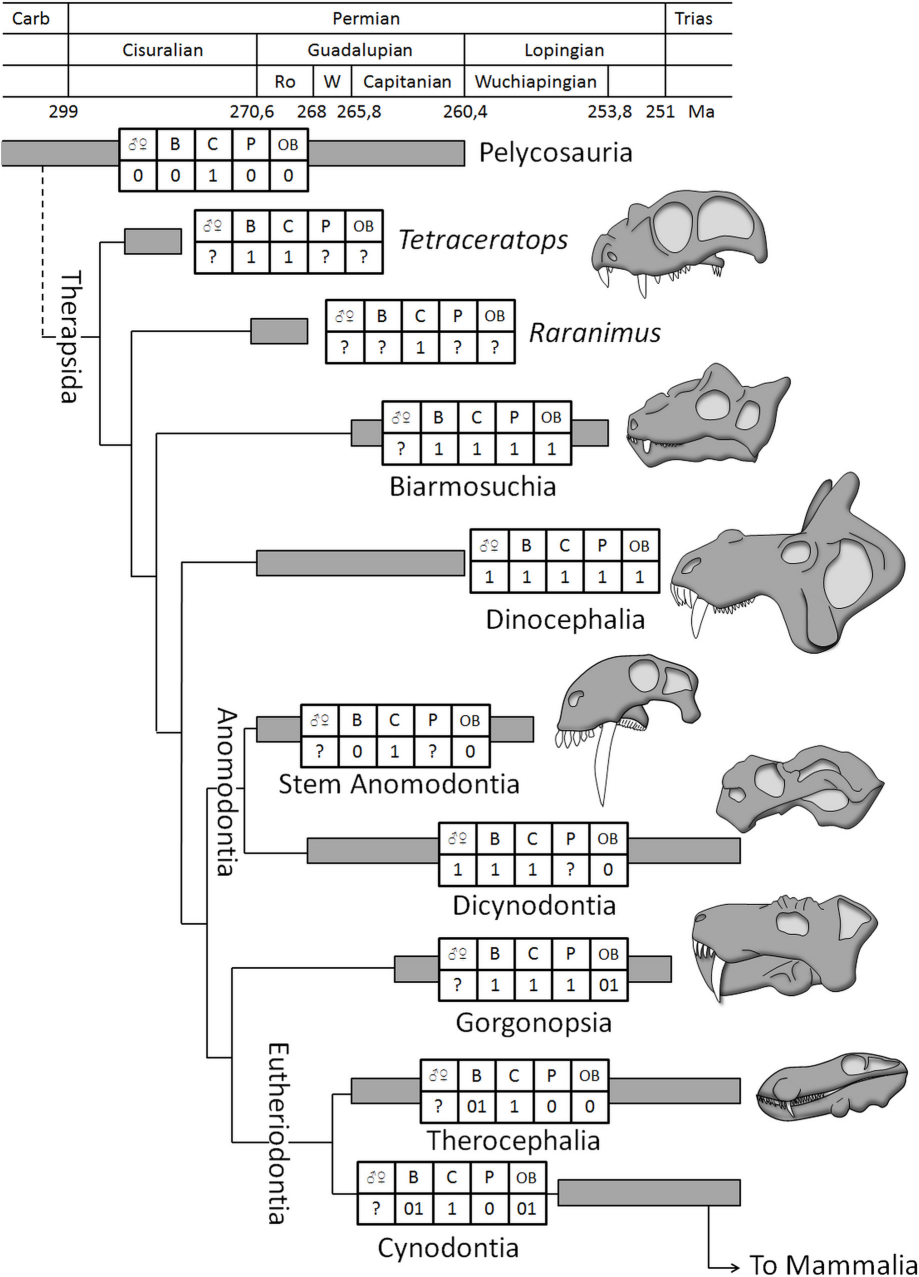
Distribution of characters linked to sexual display in non-mammalian therapsids. Abbreviations: 0, character absent; 1, character present; 01, character documented in some taxa only; ♀♂, sexual dimorphism; B, cranial boss(es); C, large canine; OB, ossified braincase; P, skull pachyostosis. Phylogeny and dates after [1, 19, 88, 92, 93].

Haughton ([40], Fig 7) depicts the parietal foramen of *Choerosaurus* in a posterior position similar to that of dinocephalians and biarmosuchians (i.e. on the caudal margin of the skull roof). As stated above, a caudal shift of the parietal foramen prevented this opening from weakening the fronto-parietal shield and appears to be associated with the re-orientation of the braincase in dinocephalians (Fig 4C and 4E). Following the same logic the apparently caudal position of the parietal foramen in *Choerosaurus* supports head-butting behaviour. However our CT study reveals that the region of the parietal foramen is not preserved in SAM-PK-K 8797 (Fig 1). Possibly the specimen was broken subsequent to the description of Haughton [40], or maybe Haughton misinterpreted the structure of this region of the skull because it is crushed (S1 Video). As no other description exists, Haughton’s assertion that the pineal foramen was located in a caudal position, though relevant for this discussion, is impossible to ascertain. We prefer to remain cautious and not to draw any conclusions about this character.

Sutures are also of importance to understand the function of cranial outgrowths. It has been suggested that an interdigitated suture would absorb more energy and thus have a greater tensile strength than a straight suture [72]. Thus, the presence of an interdigitated suture at the level of a structure suspected to be a weapon would support that hypothesis. This pattern is not helpful here since the parietal-parietal and frontal-frontal sutures which are located in the mid-line of the fronto-parietal shield in *Moschops* all appear straight rather than interdigitated on CT scan images. The same condition is observed for the maxilla-premaxilla and the dentary-splenial sutures in *Choerosaurus*, which form a close relationship with the maxillary and mandibular bosses respectively (Figs 2A–2C, 2J–2L and 5B–5D).

In the light of the features discussed above, there is not definitive evidence in *Choerosaurus* for the utilization of the cranial bosses as a weapon for high energy combat. The skull is too lightly built and the structure of the maxillary boss is weakened by the foramina for the maxillary canal. The boss on the mandible was possibly strong enough to support impacts, but given the evidence in hand, direct head to head fights as depicted in Fig (6A and 6B) appears unlikely. Flank butting appears more possible (Fig 6C) as it would lead to less cranial injury and is thus less stressfull for the skull [73]. This mode of conflict implies adaptations of the chest to resist shock [44, 56] that cannot be assessed in *Choerosaurus* as the post-cranial skeleton is not preserved.

### Did the *Choerosaurus* bosses function for display behaviours?

Another possibility is that the bosses were used for display during ritualized sexual or intimidation ceremony. In this respect, the innervation of the maxillary boss suggests a role in tactile recognition. Vascularized and innervated skin and integumentary cornified structures can be very colorful, especially in birds (e.g. Eurylaimidae, Casuariidae, Numididae, Ramphastidae) and are used for visual communication and sexual display [44, 74, 75]. As a consequence, if the bosses of *Choerosaurus* were not utilised for fighting, they may have been linked to sexual behaviour in other ways. Both interpretations are not mutually exclusive, as a natural weapon can also be efficiently used for display in extant animals [45, 73]. Indeed, an efficient fighting structure is also intimidating, and animals gain more fitness by not engaging in a fight and rather displaying their weapon to scare their opponent and to seduce mates [44, 73, 76]. An injury received through combat during or just before the reproductive season can result in a dramatic drop in reproductive success, and accordingly an intimidating stance rather than combat is often prefered [44, 73, 76]. As animals seldom engage in severe rutting combat, the primary function of a cranial ornament is likely to be for display, irrespective of its suitablility for fighting [44, 73, 76]. Accordingly the skin or conified sheath covering the cranial bosses of *Choerosaurus* likely functioned as a display tool rather than as a weapon of combat.

### Sexual selection in Therapsida

With its conspicuous osseous cranial ornaments, *Choerosaurus dejageri* may have engaged in intraspecific competition (either during display contests, sexual display, and/or low energy fights). This report thus extends the record of such cranial bosses to eutheriodonts and indicates the potential importance of sexual selection in the group of non-mammalian therapsids on the direct lineage toward mammals. If used for sexual or intimidating display, it could be expected that the cranial bosses of *Choerosaurus* would be sexually dimorphic. Because *Choerosaurus dejageri* is represented by only the holotype it is not currently possible to determine whether these structures were dimorphic or not. If the species was dimorphic, individuals lacking the bosses or having relatively smaller bosses (presumably females), could possibly be found amongst known specimens of Lycideopidae. Indeed, sexually dimorphic species have often been split into different but closely related taxonomic units (e.g. [35, 77]). As sexual dimorphism has barely been studied in eutheriodonts, sexually selected traits may have been more widespread among them than previously thought.

#### Supported cases of sexually dimorphic cranial appendages

The only therapsid species that have been demonstrated to have been sexually dimorphic are amongst the Dicynodontia (e.g. *Placerias* [39], *Pelanomodon* [22], *Aulacephalodon* [33], *Diictodon* [34], *Cistecephalus* [36], *Lystrosaurus* [35] and maybe *Digalodon* [37]). Dicynodont are know to have had a cornified beak and many species have rugose nasal and/or supraorbital bosses and ridges that are consistent with the presence of a keratinized covering or projecting nasal horns [69] that are likely to have been sexually dimorphic and involved in intraspecific combat [20–22, 39, 78, 34, 36]. Though it does not have prominent bosses, the skull of *Diictodon* displays other dimorphic characters, such as the absence of tusks in the presumed female individuals and the presence of a pineal boss, which possibly protected the pineal eye during intraspecific combat in the presumably male individuals [34]. Amongst extant mammals, cranial outgrowths and hornlike structures are used for display and combat, and are involved in the ranking of individuals during the rutting period [44, 45]. A high rank ensures access to mates or to territories to find resources and as a result the presence of cranial outgrowths is often associated with a hierarchical animal society [44, 45]. Accordingly dimorphic cranial outgrowths and hornlike structures found in dicynodonts and dinocephalians may constitute indirect evidence for gregariousness [38] as has been suggested for some dicynodont using other lines of evidence [8, 79].

Apart from the above mentioned cases, and despite the wealth of fossil therapsids from the Karoo Supergroup [1], sexual dimorphism has not undergone detailed investigation in most therapsid groups. Nevertheless, there is a large and growing body of evidence that structures associated with sexual selection (e.g. enlarged canines or cranial ornementations for sexual and intimidating display, or horn-like structures for intraspecific combats) and therefore sexual selection, played a crucial role in the evolution of Therapsida, pointing out that complex reproductive and social behaviours were a major component in the origin of mammals [8, 9, 15, 34, 38, 79].

#### Other, possibly sexually selected, cranial appendages

Amongst Permo-Triassic Eutheriodontia (Therocephalia and Cynodontia), which are mostly represented by carnivorous species, cranial ornamentation is limited to mainly enlarged canines [1, 3]. Karenitid therocephalians have symmetrical “platforms” or boss-like swellings on the postero-ventral corner of the dentary bone, in the same position as the mandibular boss of *Choerosaurus*, which have been interpreted as an adaptation to hear ground-borne sounds [42]. As they occupy the same place as in *Choerosaurus*, these structures may have in fact played a role in intraspecific selection. The therocephalians *Euchambersia* [80] and *Ichibengops* [43] have facial fossae and grooves that may have accommodated a modified gland. A venomous gland is the prefered interpretation for *Euchambersia* [1, 43], but alternative hypotheses have been proposed, such as a salivary gland [80, 81], a salt gland [82], or a scent gland that may have functioned in territorial olfactory marking in a way similar to that of many cervids [44, 83]. Among cynodonts, the traversodontid cynodont *Protuberum cabralensis* displays a thickened skull bearing several symmetrical bony swellings on the snout, zygomatic arch, and above the orbit, an important level of ossification of the braincase, as well as pachyostotic ribs [84]. These features were interpreted as a defense mechanism or as an adaptation for burrowing [84] but they are also consistent with head and flank butting (see discussion above). It is still not clear if these characters are dimorphic. Indeed, sexual dimorphism in cynodonts has only recently been suggested for the first time in *Galesaurus* and could possibly have been present in more cynodont taxa [85].

Dimorphism in gorgonopsians has never been addressed, but many gorgonopsians have a pineal boss, a feature that has been recognized as a sexually dimorphic trait in *Diictodon feliceps* [34]. Gorgonospians also have hypertrophied sabre-toothed canines, a feature that may have played a role in sexual display [9, 86]. In *Gorgonops*, *Lycaenops*, *Suchogorgon*, and *Alrausaurus* and the Rubidgeinae *Leontosaurus*, *Prorubidgea*, *Rubidgea*, and *Dinogorgon* the braincase is partially ossified by the orbitosphenoid [28, 86]. Many genera, such as *Suchogorgon*, *Dinogorgon*, *Clelandina*, and *Rubidgea* display a pachyostosed skull and supraorbital outgrowths [28, 87] which suggests that the head could have been involved in fighting during intraspecific competition. *Dinogorgon* and *Rubigea* are also remarkable as they bear a flattened dentary boss on the ventro-caudal margin of the dentary that is reminiscent of that in *Choerosaurus* and karenitid therocephalians [87]. Finally, as in biarmosuchians and dinocephalians, the gorgonopsian *Suchogorgon* displays an abundance of oblique vessels and neuro-vascular canals in its supraorbital horns [28], which are indicative of the presence of a cornified covering [28, 69].

The adaptation of the dinocephalian skull to intraspecific combat is now well established (see the discussion above). In addition, the basal dinocephalian *Estemmenosuchus* is noted for its expanded and horn-like cranial outgrowths that may have played a role in sexual display, which is supported by the possibility that the size of the cranial horns would be sexually dimorphic in Estemmenosuchids [28, 60–62, 77].

Among biarmosuchians, the burnetiamorphs have bosses and a degree of pachyostosis of the braincase, features that are well suited for intraspecific combat and display (see above). Unlike more basal biarmosuchians, the maxilla, cranial vault, supraorbital boss and zygomatic arch of most burnetiamorphs are pachyostotic [1, 28, 30–32, 63–67]. Data on the ossification of the braincase in biarmosuchians indicates that the sphenethmoid complex is well ossified and extends posteriorly, leaving only a small non-ossified space between the sphenoidal and otic region of the braincase (the metopic fissure for cranial nerve IV and V and the medial cerebral vein), as in dinocephalians [86, 88]. The presence of this ossification of the sphenethmoid complex is known in the bunetiamorphs *Lophorhinus* and *Proburnetia* as well as in the derived non-burnetiamorphs *Herpetoskylax* and *Ictidorhinus* [67, 88]. The basal biarmosuchian *Hipposaurus* also had an ossified orbitosphenoid, yet not completely enclosing the braincase since a large space separated the otic and the sphenoidal complexes [24]. A similar condition is also present in the burnetiamorph *Proburnetia* [28].

All therapsids, including the Biarmosuchia, Dinocephalia (except for derived tapinocephalids), *Raranimus* and *Tetraceratops*, as well as most pelycosaurs exhibit enlarged canines that could have served for intraspecific display and combat [1, 3, 9, 19, 89]. *Tetraceratops* also displays conspicuous cranial bosses similar to those of burnetiamorph biarmosuchians [19]. The large sail on the back of some pelycosaurs may have played a role in display [90], but Romer and Price [91] found little differences in sail size and morphology between male and female individuals of *Dimetrodon limbatus*, and no pelycosaur bear any cranial ornament.

This overview demonstrates the parallel evolution of prominent cranial outgrowths, pachyostosis and enlarged canines in therapsids, particularly in the basal forms such as the Biarmosuchia, Dinocephalia, *Tetraceratops* and *Tiarajudens*, and strongly suggests that complex behaviours associated with sexual selection (sexual display, combat between individuals of the same sex, intra- and interspecific recognition, fighting for mates and to secure territories) and sexual dimorphism were already present at the root of the therapsid clade, as early as the early Permian, and may even have an older origin among the more basal pelycosaurs (Fig 7). This implies that complex intraspecific relationships and sexual behaviour comparable to that of the most derived dinosaurs [17, 18, 45, 47] and mammals [44, 45] were already a feature of basal therapsids more than 100 million years earlier. Under this hypothesis the trend toward tooth differentiation into an enlarged canine and a pre- and post-canines dentition is a remarkable consequence of and evidence for the origin of sexual display in the ancestry of mammals.

## Concluding Remarks

The finding that *Choerosaurus dejageri* has cranial bosses that served for intraspecific agonistic behaviour, such as display or perhaps combat, extends the record of such structures to eutheriodonts and shows that complex behaviour (e.g. ritualised display toward rivals, intimidating ceremony and/or combat to secure mates and territories) was a more general feature of therapsids than previously thought. Sexual selection was probably an important component in the evolution of therapsids and mammaliformes. Further studies on sexual dimorphism among therapsids would address this hypothesis. The importance of sexual selection would account for the independant evolution of cranial bosses, horns and outgrowths numerous times in at least five therapsid lineages during the Permian, i.e. in burnetiamorph biarmosuchians, tapinocephalid dinocephalians, rubidgine gorgonopsians, the therocephalian *Choerosaurus* and in several dicynodont genera (Fig 7).

## Supporting Information

S1 Video Movie of the aligned CT slices of *Choerosaurus dejageri* SAM-PK-K 8797. Scale bar: 10mm.
(AVI)

S2 Video Segmentation of the teeth of *Choerosaurus dejageri* SAM-PK-K 8797. Maxillary teeth are in yellow, premaxillary teeth are in green and dentary teeth are in purple. Scale bar: 8mm.
(AVI)
